# A Case Series of Penile Skin Grafting in Children

**DOI:** 10.1055/s-0040-1716525

**Published:** 2020-10-21

**Authors:** Lin Qiu, Xuan Zhang, Yan Liu, Yuexian Fu, Xingang Yuan

**Affiliations:** 1 Department of Burns and Plastic Surgery, Children's Hospital of Chongqing Medical University, Chongqing, China; 2 Ministry of Education Key Laboratory of Child Development and Disorders, Key Laboratory of Pediatrics in Chongqing (CSTC2009CA5002), Chongqing International Science and Technology Cooperation Center for Child Development and Disorders, Children's Hospital of Chongqing Medical University, Chongqing, China

**Keywords:** pediatric, penis, skin grafts, lymphedema

## Abstract

Pediatric penile skin grafting is rarely performed. We present a case series of four pediatric patients receiving skin grafting due to the loss of penile skin. The four boys were followed up for 1 to 5 years. One full-thickness skin graft and three split-thickness skin grafts (STSGs) survived well with low Vancouver scar scale scores. One boy gradually developed lymphedema of the distal foreskin and underwent a second preputioplasty. He presented with normal erectile function and did not experience any pain. We propose thick STSGs as the most appropriate choice for pediatric penile skin reconstruction. Lymphedema of the foreskin is an important long-term complication of penile skin grafting.

## Introduction

Penile congenital abnormalities or traumas require adequate skin coverage for reconstruction. 1 Local flaps are preferred by surgeons, but they are not always available. 1 Alternatively, full-thickness skin grafts (FTSGs), split-thickness skin grafts (STSGs), and artificial dermis implants may be considered. The strategy is chosen depending on the location and size of the defect. Several reports were published on adult diseases requiring penile skin grafts. 2 However, only a few reports exist on penile skin grafts in the pediatric population, as diseases that cause severe penile tissue loss appear to be rare in children. Pediatric surgeons encounter hypospadias-related complications and penile skin deficiency but often prefer to reconstruct the urethra with a tabularized (or on lay-staged) buccal mucosal graft and cover the free graft with available penile skin. 3 Hence, pediatric penile skin grafting is rarely performed. We present a case series of four patients who underwent penile skin grafting at our department from 2012 to 2019.

## Case Report

### Cases 1 and 2

**Fig. 1:**
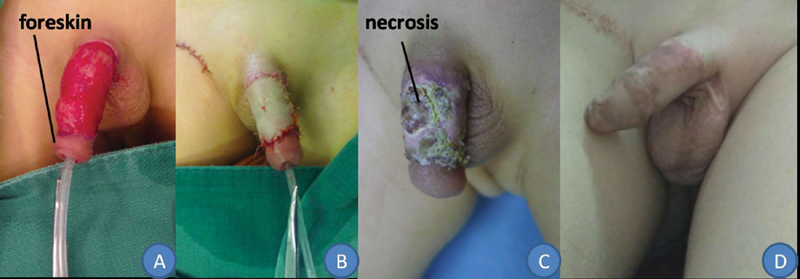
A 4.11-year-old boy suffered a high fall-related penile injury and presented with tearing of the penile skin, loss of the penile shaft skin, and retention of the front of the foreskin ( **A** ). The boy received a full-thickness skin graft (FTSG) ( **B** ). The FTSG survived almost completely at 10 days after surgery, and the small area of skin necrosis healed after changing of the dressing ( **C** ). The scar was slightly hyperplastic and hyperpigmented 8 months after surgery ( **D** ).

A 4.11-year-old boy and an 8-year-old boy suffered a high fall-related penile injury. The 4.11-year-old boy was treated 8 hours after injury. The defect area measured 3.0 × 2.5 cm. He received a FTSG after debridement because the time after injury was relatively short and the penile wound surface was relatively clean ( Fig. 1B ). Full-thickness skin was harvested from the lateral inguinal region. The inguinal donor wound was sutured. The dressing and the catheter were removed 10 days after the operation.

**Fig. 2:**
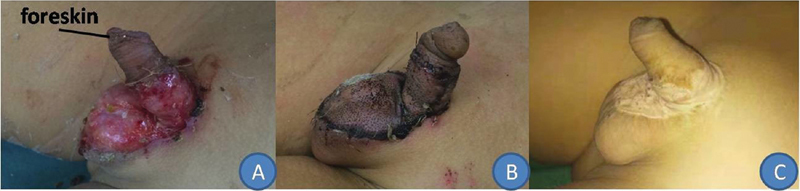
An 8-year-old boy suffered a high fall-related penile injury and presented with loss of the penile shaft skin and part of the scrotal skin, and retention of the front of the foreskin ( **A** ). The split-thickness skin graft survived completely as observed 7 days after surgery ( **B** ). The scar was noted to be flat and hypopigmented 3 years after surgery ( **C** ).

The 8-year-old boy was admitted to the hospital 24 hours after injury. The defect penile skin area measured 2.0 × 2.8 cm, and the defect scrotal skin area measured 3.0 × 3.5 cm. He received debridement and an artificial dermis implant (PELNAC, GUNZE Corporation, Japan) during the primary surgery, followed by a thick STSG 14 days after surgery ( Fig. 2B ).

### Case 3

**Fig. 3:**
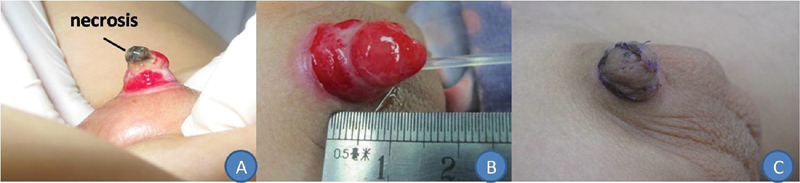
A 6.6-year-old boy suffered an electric cautery ritual circumcision-related penile injury and presented with loss of the penile skin and the glans penis ( **A** ). After debridement ( **B** ), the split-thickness skin graft survived completely as observed 1 month after surgery ( **C** ).

A 6.6-year-old boy, who sustained an electric cautery circumcision-related penile injury, was transferred from the urology department to the burns and plastic surgery department 10 days after injury. The defect area measured 1.5 × 2.5 cm. After debridement, a thick STSG was performed ( Fig. 3B ).

### Case 4

**Fig. 4:**
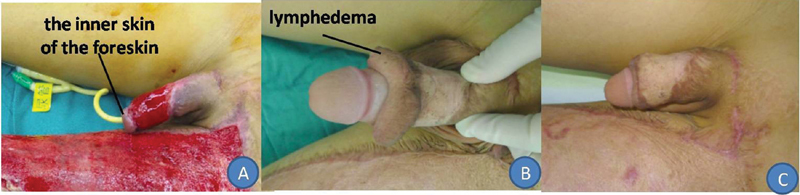
A 12.5-year-old boy suffered a traffic accident-related penile injury and presented with tearing of most of the penile skin and part of the left thigh skin, and presented with retention of the inner side of the foreskin ( **A** ). The split-thickness skin graft survived completely; however, lymphedema of the distal penile skin had developed 1 year after surgery ( **B** ). After foreskin plastic surgery, the scar was observed to be flat and hypopigmented ( **C** ).

A 12.5-year-old boy, who suffered a traffic accident-related penile injury, received primary suturing at another hospital. The penile skin necrosis area measured 2.2 × 3.1 cm, and the left thigh necrosis skin area measured 38 × 15 cm. He was transferred to our department 10 days after injury, and he received debridement and an artificial dermis implant (acellular dermal matrix) during the primary surgery. After 14 days, the acellular dermal matrix was removed, and a 0.2-mm STSG was placed on the granulation tissue wound ( Fig. 4B ).

This STSG was harvested from the scalp using a Zimmer electric dermatome. The donor site was covered with antibiotic dressing and healed naturally after 2 weeks. The penile dressing and the catheter were removed 7 days after STSG placement.

A Foley catheter was inserted and retained throughout the treatment. We used the dressing for hypospadias surgery as a reference to design the skin graft dressing. On the inner side, a mesh-like lipid hydrogel dressing (UrgoTul, Laboratories URGO, France) was evenly applied to the grafted skin so as to form a moderately tight sheath and to avoid dressing adhesion. An antibiotic ointment (mupirocin) was applied to the second layer, and the outer layer was wrapped with an elastic dressing. The graft was applied tightly to the wound surface, and the absence of dissolution or scabbing was considered to indicate good skin graft survival. The Vancouver scar scale (VSS; Baryza and Baryza, 1995) was used to evaluate the scar quality in all patients. The scale consists of the following variables: vascularity, pliability, height, and pigmentation ( Supplementary Table 1 ).

The three thick STSGs survived completely, with no signs of dissolution or scabbing ( Figs. 2 , 3 , and 4C ). The FTSG survived almost completely, with a small area of skin necrosis healing remaining after the dressing was changed ( Fig. 1D ). The 12.5-year-old boy gradually developed lymphedema of the distal foreskin ( Fig. 4B ), and he received prepuce plastic surgery 6 months after skin grafting ( Fig. 4C ). He reported that he could achieve an erection and experienced no pain during urination. All patients were able to urinate easily. The VSS scores of these four patients are presented in Supplementary Table 1 . Scar vascularity and pliability in all four patients were close to those of normal skin. The scars after STSG were hypopigmented and flat, whereas the scar after the FTSG was hyperpigmented, showing slight hyperplasia (< 2 mm). All parents were satisfied with the appearance and function of the penis.

## Discussion

Some adult diseases lead to severe penis tissue loss and require skin grafting, for example, infection, trauma, burns, malignancy, skin disorders, and primary lymphedema. 4 However, these conditions are rare in children. Nevertheless, we treated four children over the past 8 years whose penile skin defects were caused by trauma. In a pediatric hypospadias review, only 23 patients underwent FTSG, and 4 patients received STSG, among 215 patients; the 4 patients experienced a complete graft take, but secondary contraction and ulceration were observed and associated with sexual activity. 5 Thus, patients with hypospadias or “hypospadias cripples” requiring skin grafts are also relatively rare.

An ongoing debate exists about the optimal skin type to be used for penile skin grafts. A greater amount of dermis is included in FTSG than in STSG; therefore, some important distinctions in the nature and potential uses of the two types of grafts should be borne in mind. 6 For FTSG exhibiting a greater metabolic demand and requiring a well-vascularized recipient site, their survival is more uncertain. In contrast, STSGs demonstrate a lower metabolic demand and require less ideal conditions for survival; therefore, STSGs exhibit a much broader application range than FTSGs. 7 8 9 10 FTSGs display a higher degree of primary contraction, which is the recoil occurring immediately after the skin graft is harvested and is related to the amount of dermis elastin in the tissue. FTSGs may lose up to 40% of their surface area, whereas STSGs typically lose only up to 10%. 11 Over time, FTSGs exhibit a tendency to resist secondary contraction, which refers to the contraction of a skin graft after healing. Once secondary contraction ends, FTSGs tend to stretch and grow with the individual, whereas STSGs do not tend to expand, 11 which may cause contraction or scarring.

As the penile skin is thin, hairless, and flexible, and it undergoes a substantial change in size with penile erection, some researchers suggested FTSG as the preferred choice for penile skin graft. 12 13 Chertin et al 14 reported penile STSG in 17 children and adolescents, with 94% graft take, with 6 sexually active patients reporting normal sexual intercourse and sensation; none of the patients experienced shrinkage of the STSG. Alwaal et al 13 reported a successful outcome for genital STSG in 52 out of 54 adult patients, with maintained or improved erection, normal voiding, good cosmetic outcome, and normal mobility. Black et al 9 reported penile STSG in nine adult patients, and all experienced a complete graft take. A satisfactory cosmetic outcome was obtained at a mean follow-up of 6 months, and erectile function and ejaculation were preserved in potent patients. In our four patients, the three STSGs showed complete graft survival, and the FTSG showed almost complete survival, with a small area of skin necrosis healing after changing of the dressing. The small area of skin necrosis may have been caused by a contaminated wound or/and be due to the change in penile size with erection, which caused poor metabolism and blood supply in the FTSG. All boys were able to urinate easily, and the 12.5-year-old boy could achieve an erection without experiencing any pain. Based on the above-listed observations and the results from our own study, thick STSG appears to be the most appropriate choice for penile skin reconstruction.

Long-term complications of penile skin grafting should be borne in mind. The 12.5-year-old boy who received an artificial dermis implant (acellular dermal matrix) and a thick STSG gradually developed lymphedema of the distal penile skin ( Fig. 4B ) and required extra foreskin plastic surgery. Diaz et al noted 15 that lymphedema may develop if any excess distal penile skin is not excised, and limiting the amount of mucosal collar or consider direct anastomosis to the glans is prudent. When loss of the proximal penile skin occurs, the tendency is to preserve any viable distal skin. However, when loss of the proximal penile skin occurs, the proximal dartos layer is usually lost, resulting in disruption of lymphatic drainage of the distal penile skin. The native skin should be resected up to the level of the glans to avoid a ring of lymphedematous skin developing around the glans. If skin loss is not circumferential, leaving the native skin in place is preferable, especially if several days of observation have not resulted in any increase in skin edema. 6 The 12.5-year-old boy received an artificial dermis implant (acellular dermal matrix) during primary surgery, and the 8-year-old boy received an artificial dermis implant (PELNAC, GUNZE Corporation, Japan) during primary surgery. However, only the 12.5-year-old boy developed lymphedema. The PELNAC grows on the wound bed and forms granulation tissue, with a structure similar to that of the dermis. However, the acellular dermal matrix is a short-term onlay, which covers the wound bed and stimulates the growth of granulation tissue. The matrix was eventually removed. Whether the implant was the cause of the lymphedema remains unknown, and so, more cases and studies are required.

## Conclusion

In conclusion, pediatric penile skin grafting is a rare procedure. Based on our experience and observations published in the scientific literature, thick STSG appears to be the most appropriate choice for pediatric penile skin reconstruction. Lymphedema of the foreskin is an important long-term complication of penile skin grafting.

## Notes

New Insights and the Importance for the Pediatric Surgeon Skin grafting of the pediatric penis is not performed frequently. The aim of this study was to put forward some thought-provoking observations about penile skin grafting in children after considering our study results and other literature.
