# Supplementary material for: A Case Series of Penile Skin Grafting in Children
Source: European J Pediatr Surg Rep. 2020 Oct 21;8(1):e77–80. doi: 10.1055/s-0040-1716525 (PMC7577789; doi:10.1055/s-0040-1716525)
Supplement: Supplementary file 1 — Supplementary Material [file 10-1055-s-0040-1716525-s200522cr.pdf]

**Supplementary Table 1** The Vancouver scar scale scores of the four patients

| | Pigmentation<br>0—close to normal<br>1—hypopigmented<br>2—mixed<br>3—hyperpigmented | Pliability<br>0—close to normal<br>1—supple<br>2—yielding<br>3—firm<br>4—banding<br>5—contracture | Height<br>0—flat<br>1—2 mm<br>2—2–5 mm<br>3—5 mm | Vascularity<br>0—normal<br>1—pink<br>2—red<br>3—purple | Vancouver scar scale score |
|---|---|---|---|---|---|
| A 4.11-year-old boy | 2 | 0 | 1 | 0 | 3 |
| A 6.6-year-old boy | 1 | 0 | 0 | 0 | 1 |
| An 8-y-old boy | 1 | 0 | 0 | 0 | 1 |
| A 12.5-year-old boy | 1 | 0 | 0 | 0 | 1 |
